# Generation and applications of cardiac spheroids

**DOI:** 10.1038/s44385-025-00024-y

**Published:** 2025-06-10

**Authors:** Dana Idais, Christopher D. Roche, Georgia Kalogianni, Liudmila Polonchuk, Carmine Gentile

**Affiliations:** 1 Cardiovascular Regeneration Group, Heart Research Institute, Sydney, NSW Australia; 2 School of Biomedical Engineering/FEIT, University of Technology Sydney, Sydney, NSW Australia; 3 Sydney Medical School, The University of Sydney, Sydney, NSW Australia; 4 Roche Pharma Research and Early Development, Roche Innovation Centre Basel, F. Hoffmann-La Roche Ltd., Basel, Switzerland

**Keywords:** Tissues, Cardiovascular biology

## Abstract

Cardiac spheroids (CSs) offer valuable insights into the fundamental aspects of cardiac biology as they model molecular, cellular, and extracellular features typical of the myocardium. This review introduces current engineering methods for CS generation and their applications. Commonly referred to as “mini hearts”, their applications include disease modelling, drug and toxicity screening, and personalised therapeutics in cardiac regenerative medicine.

## Introduction

Cardiovascular diseases (CVDs), including myocardial infarctions (MI) and strokes, are among the leading causes of death globally. As of 2019, CVD’s have accounted for approximately 85% mortality rates following their onset^1^. Injury resulting from CVD in adults results in fibrotic scarring and hypertrophic remodelling of cells surrounding undamaged myocardium, contributing to increased tissue density and consequent reduction in elasticity and flexibility within the localised injured sites. This limited regenerative capacity is a significant barrier to complete functional cardiac recovery and is largely attributed to the loss of cardiomyocytes (CMs). CMs are key cellular constituents of the myocardium responsible for muscular function and contractility. Contrary to their role at birth, where they undergo avid growth and exhibit regenerative properties, in adulthood, CMs display reduced regenerative capacity and are unable to compensate for the substantial cellular losses and contractile demands resulting from MI or other cardiac injuries^2^.

Three-dimensional (3D) cell cultures of cardiac cells represent a rapidly evolving multidisciplinary field with considerable promise for transforming clinical and research paradigms relating to cardiac and regenerative medicine. Compared to classical 2D cell cultures, 3D cultures closer approximate the crucial biochemical and physiological qualities that mimic a functional heart, hence offering a supportive niche for cell-cell interactions, conductivity, self-organisation, and differentiation^3^. The spherical microtissues generated by this culture method are referred to as cardiac spheroids (CSs) or organoids (cardioids), which can be generated using primary- or induced pluripotent stem cell-derived (hiPSC) cardiomyocytes alone, or in conjunction with other cardiac present cells; including pre-differentiated cardiac fibroblasts (CF), which contribute to components of gap junctions and ECM in the developing myocardium, endothelial cells (EC) and/or smooth muscle cells (SMCs), which promote vascularisation and CM maturation. Various cellular compositions have been shown to enhance spheroid survival and better represent the physical structure, conductivity and signalling pathways of native tissue^4–8^.

Briefly, spheroid formation occurs through spontaneous cell aggregation and binding of cell surface integrin receptors with components of the extracellular matrix (ECM) (*i.e*., laminin, fibronectin, and collagen) to promote cell-cell contact and E-cadherin accumulation on the surface of cells. This interaction facilitates strong intercellular signalling and promotes spheroid compaction. Contrasting to 2D cultures, CSs provide better accuracy in emulating cellular functions and signalling pathways of target tissues by their ability to form cell-cell and cell-matrix interactions^9^. However, this can be limited by the biocompatibility of the materials used for bioprinting, or simply by a spheroid’s microenvironment, which can promote hypoxia at a spheroid’s core that is surrounded by an inner layer of quiescent cells and an outer layer of proliferative cells in consequence of limited oxygen and nutrient diffusion, particularly at diameters exceeding 200 micrometres^10^.

Various studies have investigated 3D cultures using either scaffold-free methods, which take advantage of the natural self-adhesion properties of cells to form spheroids, or scaffold-based methods, where cells are seeded in an acellular or liquid-based matrix, generated using biological or synthetic-derived biomaterials, like Matrigel™ or Poly (ethylene glycol) (PEG), respectively^11^.

**Fig. 1:**
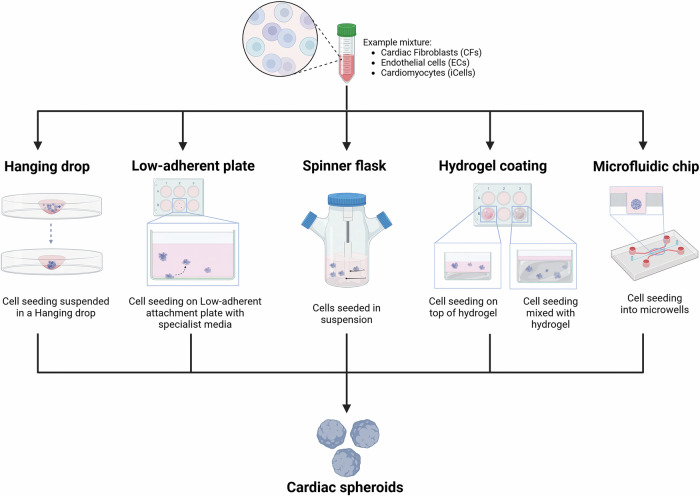
Methods of 3D spheroid generation. Diverse cell types (represented by different shades) self-organise to form compact spheroids based on the utilised technique: Hanging drops, Low adherent plates, spinner flask, mixed hydrogel with desired cell combinations either embedded over or mixed into the hydrogel and microfluidic devices (organ-on-a-chip). CFs cardiac fibroblasts, HCAECs coronary artery endothelial cells, iCells induced pluripotent stem-cell derived cardiomyocytes.

To date, CSs are receiving increasing attention within the biofabrication and regenerative spaces, with new advancements in technologies influencing methods of their generation and potential applications^10^. Common techniques of generation encompass a range of methods, including hanging drop (HD) cultures^3,7,10^, microwells^12^, rotary spinner flasks^13^, suspension in specialised media^14,15^, microfluidics^16^, and bioprinting^17–19^ (Fig. 1). These techniques are not exclusive and are being combined in innovative ways, such as through the combination of microwells and rotating devices^20,21^, or the integration of microfluidics with bioprinting technology^22^ to improve CS generation and their mass production.

In this review, we examine the potential CSs have in shaping clinical and pre-clinical cardiovascular research by introducing established methods of CS generation and highlighting their in vitro and in vivo applications in i) disease modelling; ii) drug screening and cardiac safety testing; iii) 3D bioprinting for cardiac regeneration. Topics such as future directions and emerging paradigms of this rapidly evolving field are addressed, including their vascularisation and new bioengineering approaches.

## Generation of cardiac spheroids

### Hanging drop cultures

Hanging drop (HD) cultures represent one of the most widely used approaches to spheroid generation. Traditionally, HD cultures are generated by pipetting cells in suspension onto the underside of a petri dish lid which gets inverted to promote the accumulation of cells to the centre of each drop through surface tension and gravitational force^23^. As an open culture system, this method allows optimal gas exchange to occur during incubation whilst also avoiding bubble formation. In this method, droplet evaporation can be prevented by the incorporation of a hydration chamber consisting of a sterile buffer, like PBS or water at the bottom of the plate^24,25^.

Several specialised HD plates have been developed for generating spheroids, such as Perfecta 3D® 96-well HD plates (3D Biomatrix, MI, USA), which are compatible with manual pipetting, liquid handling systems and microplate readers^6^. For high throughput CS generation, plate options offering more wells are also available such as 384-well HD plates (3D Biomatrix, MI, USA) or Elplasia™ Mpc 350 plates (Kuraray Co., Ltd., Tokyo, Japan), the latter of which, features microcavity wells, that allow generation of hundreds of spheroids per well, all exhibiting uniform conditions during experimentation^26^. In addition to dedicated HD devices, spheroids can also be generated using the inversion of ultra-low-adhesive (ULA) 96-well GravityTRAP plates (GravityTRAP^TM^, InSphero, Schlieren, Switzerland). This method combines the upward-facing low adhesive surface (microwell) method, followed by the downward-facing HD method, using the same hydrophobic polystyrene plate^27^. Others have used, hydrophobic plates with wettable areas, which cause the fluid-based cellular material to aggregate into spheroids that then get inverted, turning them into HDs^28^. Generally, these modifications (Table 1) to the HD technique enhance high throughput testing, particularly for drug screens using large libraries to expedite drug discovery.

**Table 1:** Overview highlighting examples of 3D cell culture methods and their respective commercially available products

Method	Product	Manufacturer	References
Hanging drop	Perfecta 3D®	3D Biomatrix (MI, USA)	^6^
	Elplasia™ Mpc 350 plates	Kuraray Co., Ltd. (Tokyo, Japan)	^12^
	GravityTRAP^TM^ Utra-low Attachment plates	InSphero (Schlieren, Switzerland)	^24^
Forced floating (Low-Adhesive Surfaces)	PrimeSurface® ULA Cell Culture Plates	Sumitomo Bakelite Co., Ltd. (NH, USA)	^37,38^
	AggreWell™ plates	STEMCELL Technologies (Vancouver, Canada)	^39,40^
	Nunclon™ Sphera™ 96-Well, Nunclon Sphera Treated, U-Shaped-Bottom Microplate	Thermo Fisher Scientific (MA, USA)	^7^
Agarose-micro moulds	MicroTissues® 3D Petri Dish® micro-mould spheroids	MicroTissues, Inc., (MA, USA)	^41^
Spinner flasks	Corning® Disposable Spinner Flasks	Merck (Darmstadt, Germany)	^42,44^
Microfluidics	AIM 3D Cell Culture Chips	AIM Biotech PTE. Ltd. (Singapore)	^49^

Spheroid size, influenced by cell density, is a crucial barrier that impacts organoid generation with the capacity to alter biophysical properties like pH, oxygen, nutrients, and waste removal within CSs, as well as their biological profiles at transcriptomic and metabolic levels^24,25,29,30^. Scalise et al. (2023) recently explored the relationship between the approach used to generate CSs (ultra-low attachment plates with a flat surface, HD cultures and agarose micromoulds) and cardiomyocyte differentiation. This study showed that CSs generated using HD cultures presented higher expression levels of cardiac contractile proteins, such as Myl2-7, Tnnt2 and Actc1, than those generated using ultralow attachment flat surfaces, suggesting enhanced in vitro differentiation. Additionally, smaller spheroids in diametre exhibited reduced Hif-1α expression, suggesting reduced hypoxic core formation in these spheroids^24^.

Co-culture protocols utilising HDs aim to enhance the complexity, functionality, and maturity of CMs in spheroids, through optimised cell ratios for CS generation^6^. For example, CSs generated using a 2:1:1 tri-culture ratio of human primary adult cardiomyocytes or iPSC-derived cardiomyocytes (iCells), with iPSC-derived cardiac fibroblasts (iCFs) and coronary artery endothelial cells (HCAECs) exhibit syncytial contraction and transcriptomic regulation that mimic the physiology of native myocardial tissue^6,31–33^. In two-cell type HD cultures, others use a 4:1 ratio of hiPSC-CMs: CFs to mimic healthy tissue, void of fibrosis^8^.

In addition to the optimisation of cellular compositions making up spheroids, the microenvironment in which they are cultivated is a crucial feature of consideration. Altering media composition, through the addition of methylcellulose and/or collagen, for instance, positively impacts spheroid circularity and compaction by forming tighter spheres, with optimal conditions determined by the cellular composition of spheroids^31^. Such effects are also evident with tumoral spheroids, which exhibit similar effects with methylcellulose, along with increased medium viscosity, which contributes to reducing motion-induced image blur during imaging^31,34^. While this offers imaging advantages, changes in medium viscosity remain a consistent challenge that must be optimised to the medium composition opted for, as an increase in osmolality could alter the biophysical properties of spheroids.

Other enticing features for using HD cultures include their low cost, short generation time, utility to use independent of scaffolds, and the low cell volume required per spheroid generated, a feature that is appealing in cases utilising precious samples. Though, labour intensiveness (if executed without robotic equipment), challenging media changes, and high cross-contamination risks are drawbacks currently impeding their application for mass spheroid production^35^.

### Forced floating (low-adhesive surfaces)

Liquid overlay on ultra-low attachment (ULA) plates is another widely adopted approach for CS generation due to their simplicity and low cost. Liquid overlays utilise a pre-coating technique involving an ultra-hydrophilic polymer that creates a setting in which the adhesive forces between cells are stronger than the surface they are cultured on. Therefore, enabling the free-floating cells to form aggregates of consistent size uniformity across multiple wells. Alternatively, one can also utilise uncoated polystyrene-surface plates, which possess low adhesive properties. Commercially available ULA-pre-treated plates are currently offered, like the ULA-PrimeSurface® Cell Culture Plates (Sumitomo Bakelite Co., Ltd., NH, USA) and Nunclon™ Sphera™ 96-Well, Nunclon Sphera-Treated, U-Shaped-Bottom Microplate (Thermo Fisher Scientific, MA, USA), in 96-well or 384-wells formats, making them suitable for high-throughput production. ULA plates can also be made manually using standard culture plates coated with an appropriate volume of anti-adherence solution per well^36–39^. Once plated, cells are centrifuged and incubated for 24–48 h to allow for aggregation. Moreover, like HD’s, microwell-based options like AggreWell™ plates (STEMCELL Technologies, Vancouver, Canada), which feature 400 or 800 μm sized microwells are available in 6- or 24-well options, providing vessel-based size control of the spheroids. Like the liquid overlay method, cell suspensions get aliquoted into wells, and through centrifugation, get evenly distributed into the microwells before being incubated^40,41^.

Opting for CS generation using ULA plates provides high optical clarity, allowing for utilisation of commonly available imaging platforms, like bright field and confocal microscopy. This is advantageous, as CSs in HDs tend to be unstable and prone to evaporating (due to the low medium volume required), and typically require transfer into standard plates for cell-based assays, like drug-toxicity testing^11^. Additionally, ULA plates can be used for assays requiring transient drug exposures, which in HDs cannot be achieved as the removal of added agents is not possible. While both HDs and ULA methods generate compact spheroids with smooth surfaces, the ULA method can be prone to generating multiple spheroids per well, presenting variability in CS size and shape due to fusion.

### Agarose-micro moulds

Agarose micro-moulds have more recently emerged as a reliable and reproducible tool in the generation of CSs, facilitating the consistent formation of 3D cell cultures with defined shapes and sizes. MicroTissues® 3D Petri Dish® micro-mould spheroids (MicroTissues, Inc., MA, USA) is a specific example of this approach, utilizing micro-moulds made of agarose, a biocompatible, non-toxic polysaccharide that allows cells to aggregate naturally without adhering to the mould itself^42^. The process begins by casting agarose micro-moulds that will later serve as templates for spheroid formation. Sterilised agarose is dissolved, poured into the micro-moulds, and then allowed to solidify, forming wells with controlled, repeatable geometries. The gelled agarose is then carefully removed and transferred to standard culture plates under sterile conditions. Once equilibrated with cell culture medium, cardiac cells, such as cardiomyocytes or cardiac progenitor cells, are seeded dropwise into the agarose wells. This setup promotes cell aggregation within the confined space, encouraging cell-cell interactions that lead to spheroid formation.

Agarose-based micro-moulds can be created in various well sizes (ranging from 35 to 256 as offered by MicroTissues, Inc., MA, USA), accommodating different cell densities and spheroid dimensions, which allows for flexibility in experimental design and applications. Additionally, the non-adherent nature of agarose minimises interference with cellular processes, enabling cells to self-assemble into spheroids, a feature that mimics the in vivo cellular environment more closely than traditional 2D cultures^42^. Once formed, the spheroids can be maintained and cultured in the agarose moulds or transferred to other platforms for further analysis.

Agarose micro-moulds provide a scalable, cost-effective, and reproducible method for generating CSs, making them a valuable tool in cardiac tissue engineering and the study of drug-induced cardiotoxicity. As such, they are increasingly being adopted in organ-on-a-chip platforms, where the combination of 3D cellular organization and controlled environmental conditions enhances the physiological relevance of in vitro cardiac models.

### Spinner flasks

In contrast to the static culture methods discussed, spinner flasks rely on the constant spinning of cells in a suspension of growth medium within a cylindrical container to form cell aggregates. Briefly, as well as preventing cell sedimentation, this method enables the continuous and uniform cycling of oxygen, pH, metabolites, and nutrients to cells, thereby preserving cellular viability^43,44^. Recently, a partially GMP-compliant spinner flask method generating hPSC-CM 3D aggregates demonstrated a yield exceeding 100 million CMs per 300 mL of culture medium, setting the potential for standardised CM differentiation and aggregation, both of which are crucial controls for regenerative medicine applications^43,45^.

Commercially available flasks, like the Corning® Disposable Spinner Flasks (Merck, Darmstadt, Germany) can store a wide range of liquid volumes (125, 500, 1000, or 3000 mL), providing a significantly high scalability advantage to this system over HD’s and ULA-plates. In addition, these flasks can be equipped with sampling ports as well as oxygen, temperature, and/or pH sensor probes to monitor environmental conditions in culture using marketed attachments like the Corning® Reusable Probe Insertion Fitting for Vertical Sidearm Flasks (Merck, Darmstadt, Germany). While the incorporation of such features within the dynamic environment produces more uniform and viable spheroids, fundamental challenges affecting control over spheroid density and size due to the spontaneous aggregation of free-floating cells, as well as the mechanical shear-stress by the turbulent flow (*i.e*., effects of the physiological response of cells based on rotational speed), must be accounted for when opting for this system. An alternative method that can overcome such limitations is the use of microfluidic chips which allow for better control over the cycling of nutrients, drug-screening compounds, or maturation cues of interest, as fluid-to-spheroid-size flow can be pre-calculated and adjusted to reduce shear-stress exposure and to generate more clinically robust outcomes^30^.

### Microfluidics

Similar to the spinner flask method, but in a smaller scale and therefore with limited scalability, is the use of microfluidic devices to generate CSs. Enticing features of using microfluid devices that overcome some of the challenges of the spinner flasks include precise control over spheroid size and composition based on microwell and microchannel inclusion. Recent studies have explored enhancing the survival of cardiac-derived cell aggregates by microencapsulating them in spherical beads within microfluidic chips^46,47^. Heterogenous explant-derived cardiac cells cultured directly from heart tissue biopsies have been used in this case, and ‘cocooned’ in protective nanoporous gel (NPG) capsules made of low melt agarose, human fibronectin and human fibrinogen^47^. With this approach, the microfluidic device consisted of inlets intersecting perpendicularly to allow the passing and encapsulation of premixed cells/NPG from one inlet, with mineral oil passed through the other inlet to form droplets that gel as they later pass through a cooled serpentine path^47^. The ability to adjust nozzle diameter (ranged from 30 to 50 μm) and flow pressure enabled precise control over droplet size and cell density (~300 cells/s), enhanced injectate purity (using an inlet filter), improved the amount of biomaterials used and reduced shear-stress exposure^47^. These are unique features that cannot be achieved in vortex-based encapsulation methods, which do not inherently control for droplet size and lead to low cell viability and inconsistent encapsulation yield^47^. The potential to adopt a similar microfluidic-encapsulation method for the direct generation of multi-cellular CSs is yet to be reported in the literature, perhaps additional ports will need to be included and parameters reoptimised. Nonetheless, research into this space will eventually permit large-scale spheroid generation with reduced batch-to-batch reproducibility and allow for the generation of personalised injectable spheroids for clinical application^47^.

**Fig. 2:**
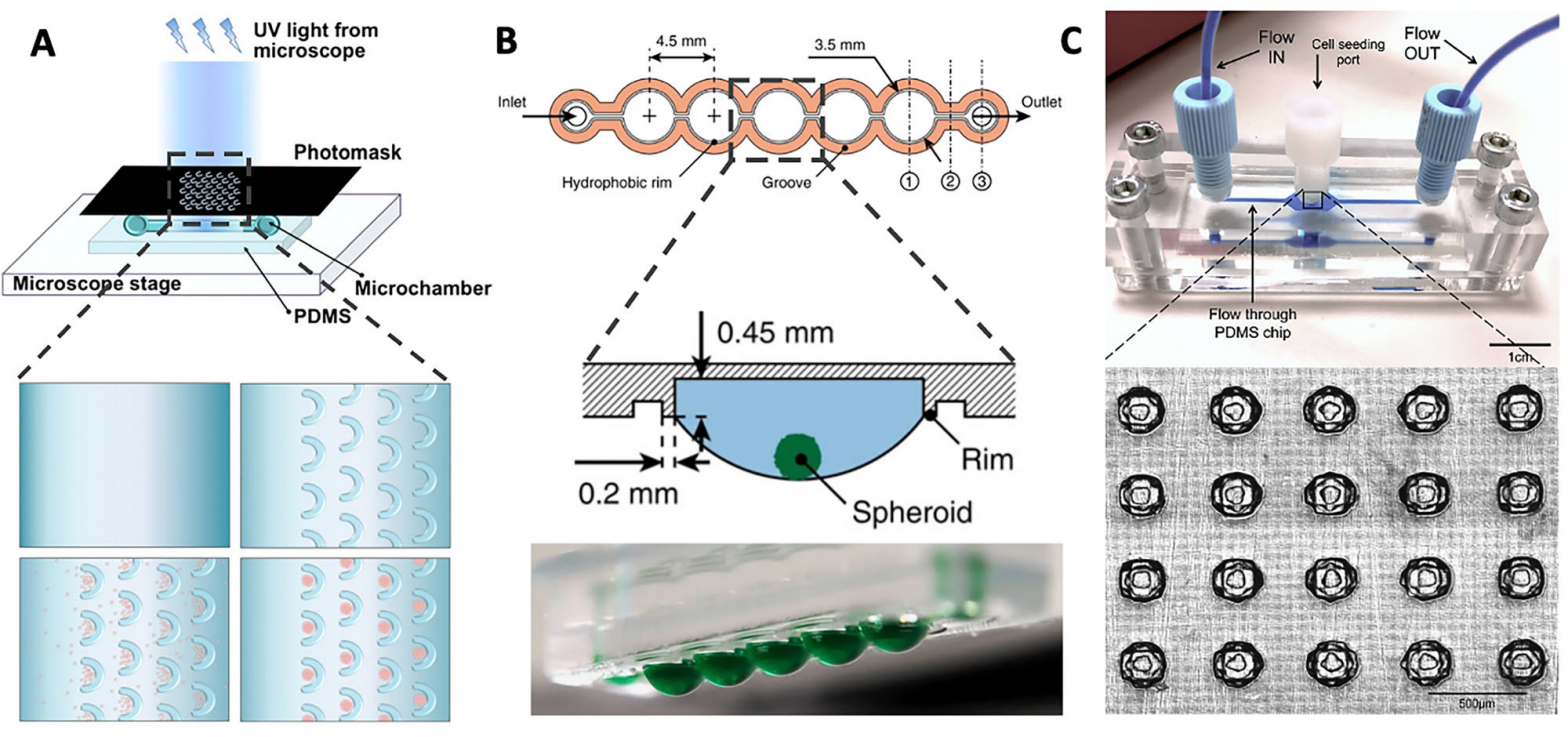
Microfluidic devices for spheroid generation. **A** Fabrication set-up of microfluidic chip with integrated U-shaped microstructures. UV light polymerizes a PEGDA solution within a PDMS microchamber, forming hydrogel structures. The sequential images show (1) pre-polymer injection, (2) hydrogel U-shaped microstructures formation, (3) cell trapping, and (4) spheroid formation (modified from ref. ^49^). **B** A “hanging-drop” microfluidic system, where spheroids are suspended and maintained by continuous nutrient flow (modified from ref. ^50^). **C** A microfluidic device with accessible wells for manual cell seeding, facilitating the generation of cardiac spheroids (modified from ref. ^51^).

Of note, microfluidic systems leverage the continuous and precise mixing, perfusion, and gradient-based control of nutrient and waste levels to establish a microenvironment closely mimicking in vivo conditions of the human heart—this can allow the generation, manipulation, and testing of spheroids within the same microfluidic process, offering significant advantages for high-throughput testing^48^. A variety of microfluidic designs have been explored for spheroid generation, including low-adherent U-shaped traps that capture cells via fluid flow. For example, one study employed a PDMS microchamber with UV-polymerized PEGDA hydrogel structures that enabled passive cell trapping and spheroid formation through fluid flow and gravity (Fig. 2A)^49^. This suggests how structural and surface modifications can enhance cell aggregation in microfluidic platforms. Alternatively, other studies explored a “hanging” microfluidic system, to produce HD-style spheroids maintained by a continuous flow of nutrient-rich media. One such platform utilises an inverted PDMS substrate, where capillary forces guide the liquid flow to form HDs that serve as spheroid compartments. This system enables precise nutrient delivery, substance dosing, and metabolic exchange between multiple spheroids, which can be interconnected in a physiological order (Fig. 2B), allowing for parallel spheroid formation and subsequent integration into complex multi-tissue networks or “body-on-a-chip” research^50^. More recently, a redesigned microfluidic system with wells accessible for manual cell seeding, producing a higher percentage of CS generation compared to perfused seeding methods has been proposed (Fig. 2C)^51^. Incorporated within the later design are independent multichannel pumps for use in end-point monitoring of drug effects as well as the use of poly(methyl methacrylate) layers in the fabrication of the device to allow on-chip fluorescence imaging for cell viability, providing yet another reduction in handling for imaging, where excessive movement tends to compromise spheroid integrity, particularly with long-term workflows^51^. Alternatively, commercial options such as the AIM 3D Cell Culture Chips (AIM Biotech, Singapore), which facilitate organotypic co-culture models, leakproof hydrogel injection as well as control over chemical gradients and flow through are readily accessible and serve as a cost-effective substitute to facilities lacking engineering capabilities^52^.

As microfluidic devices provide the ability to generate and drug-screen 3D spheroids on the same platform, some suggest that akin to the advantages of 3D cell culture models over monolayer cultures for drug testing, the flow generated through this platform is required to effectively evaluate physiological human cellular responses compared to fluid-static cultures^53^. Experimentally, the gradient-based control offered by microfluid devices provides controlled regulation and exposure of cells against the ‘unnatural’ levels of added drugs, mimicking better physiological absorption and subsequent reactions during drug screens—else bulk exposure of added factors is an intrinsic byproduct of bulk media changes in the other described methods. Moreover, issues like forced inhabitation of cells (as in 2D culture) are mitigated, thereby eliminating the effects of unnatural polarity responses between the upper and lower surfaces of matrix-adhered cells^54^.

A promising recent advancement in microfluidic fabrication involves the bioprinting of microfluidic chips featuring perfusable vascular networks and 3D cultures, addressing the need for sustained vascularization in engineered tissues^55^. Di Cio et al. produced a functional micro-vascularised on-chip cardiac model using photo- and soft lithography-based printers to initially print their microfluidic model, which was subsequently injected with vascular cells (HUVECs/pericytes at a ratio 10:1 ratio) and incubated to vascularise for four days before CS seeding. Among their findings, this study exemplifies the complex responses of spheroids to matrix remodelling, as shown by the 48-hour halt in CS beating (restored thereafter) when initially embedded in the vascularised chips compared to control spheroids grown in suspension^55^. Highlighting the direct generation of spheroids through microfluidics, one study used a vacuum-assisted microfluidic chamber to produce 3D cardiac microtissues from iPSC-CMs and rat cardiac fibroblasts within a fibrin-based hydrogel solution^56^. Once injected, the cell-hydrogel solution resulted in the aggregation and contraction of 3D cardiac microtissues. Subsequent experiments, involving injections of doxorubicin, sotalol, and verapamil into the chambers, yielded results on the same CSs produced within the unit^56^.

### Bioprinting

3D bioprinting allows for scalable, uniform, automated mass production of spheroids through additive manufacturing (AM) technologies. Typically, such technologies are coupled with biological materials and scaffolds that generate physiologically replicative constructs of target tissues. Novel biomaterial inks and printers are constantly under development to enhance cell survival and functionality within constructs and advance the manufacturing process of cardiac models. For instance, one study compared bioprinting of cardiac tissues with the REGEMAT 3D BIO V1 (Spain) and CELLINK BioX (Sweden; Blacksburg, VA, USA) bioprinters to demonstrate the effect of hydrogel compositions on CS survival^57^. Both extrusion bioprinters 3D print a biomaterial that is gradually deposited on the print bed layer-by-layer as it contacts the hot extruder surface during printing. High-resolution printing is an advantage to this form of printing, as droplets of minute volumes can be generated, although with limitations based on the narrow range of bioink viscosity required (~3.5–12 mPa.s) and the constant need to optimise gelation post-printing to sustain the construct's structural integrity.

**Fig. 3:**
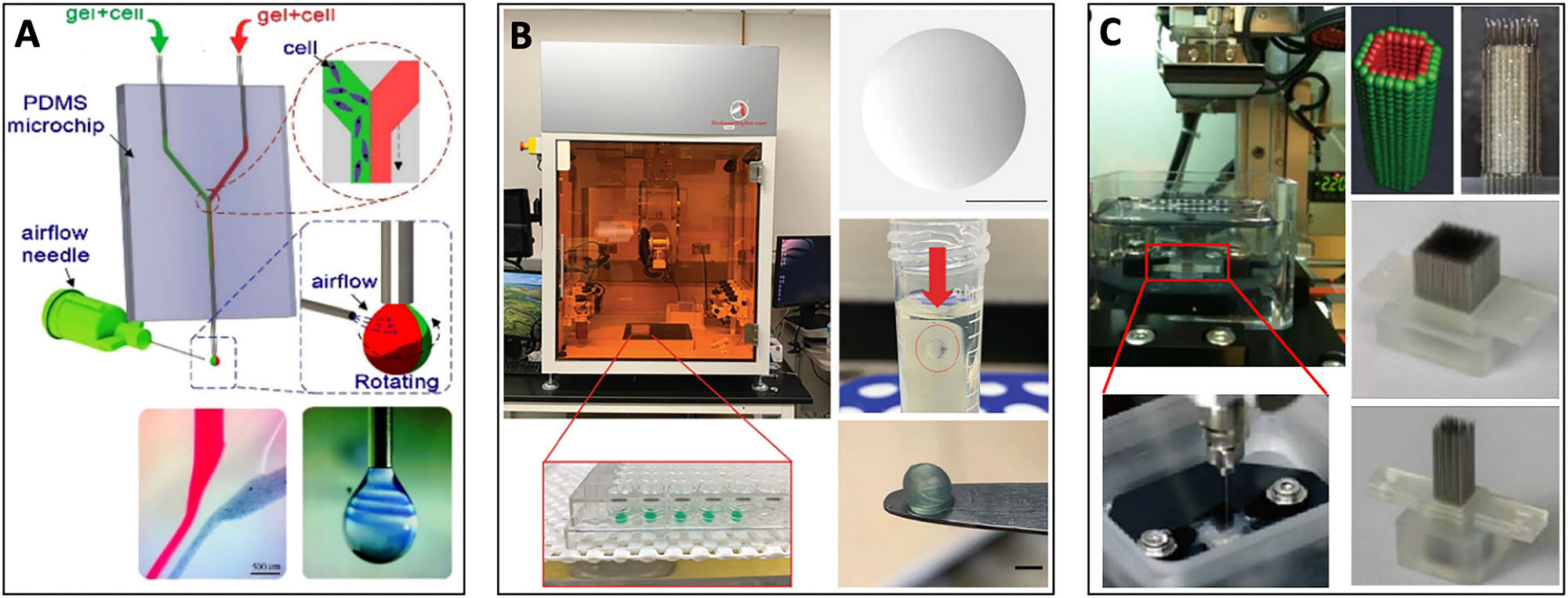
Bioprinting methods for cardiac spheroid generation. **A** A 3D bioprinter with an airflow-assisted microfluidic nozzle extrudes sodium alginate solutions, which are rotated by airflow to form spiral-shaped, cell-laden droplets (modified from ref. ^22^). **B** A hybrid method using the BioAssemblyBot (BAB) 3D printer that creates consistent spheroidal hydrogel droplets onto a 96-well plate with images demonstrating the morphology (modified from ref. ^58^). **C** Kenzan method for spheroid deposition; using microneedles to precisely deposit spheroids using the Regenova® 3D bioprinter (modified from ref. ^147^).

In the direct case of spheroid bioprinting, various methods can be employed. One study used a microfluidic nozzle, cell-laden to form a spiral-based droplet through airflow-driven rotation (Fig. 3A)^22^. In this context, gravitational pull dispenses individual spheroids into a plate of CaCl_2_ (2% w/v) as a final cross-linking step using sodium alginate-based bioink^22^. Khoury et al.^58^ used a more direct method that incorporated hybrid printing technologies, like the CELLINK BioX and the Advanced BioAssemblyBot®400 (BAB400) system which they tested using human cardiomyocytes and cardiac fibroblasts to generate spheroidal droplets (Fig. 3B). In their work, a balance between open and closed pores was reported, contributing to conclusions of improved nutrient transport and mechanical fidelity, respectively^58^.

An alternative, yet interesting method, which falls outside the definition of bioprinting but has similarities in mechanism and appeal for spheroid generation, was put forward by Munarin and Coulombe^59^ in the form of the Var J30 Bead Generator (Nisco, Switzerland). This machine was used to jet spray ‘microspheres’ of alginate into a gelling bath which were then incorporated into a scaffold used for cardiac regeneration. The microsphere diameters could be controlled and were typically 100 µm (range between 50 and 450 µm), similar in size to typical spheroids. Whilst the spheroids generated in this experiment were not cellular themselves, when engrafted onto rat hearts, they provided a scaffold for host cardiac cells to grow around. The method was not attempted with cells, but with manipulation, it could foreseeably provide inspiration for a novel jet-spray method of primary cellular spheroid generation in the future as it has been shown that even stem cells can survive bioelectrosprays and aerodynamically-assisted bio-jetting without gross cellular damage^60^.

In another approach, the Kenzan (‘mountain of needles’) method uses the Bio 3D-printer Regenova® (Cyfuse Biomedical, Tokyo, Japan) for spheroid cardiac tissue generation^61^. In this method, pre-assembled spheroids are transferred into ULA round-shaped 96-U-well plates (Sumitomo Bakelite, Tokyo, Japan), and are ultimately impaled by a robotic arm onto microneedles which provides a temporary matrix for precise deposition and spatial organisation into secondary spheroid-based tissue structures (Fig. 3C)^19^.

## Applications of cardiac spheroids

### Cardiac regeneration

The effective isolation of hPSC-CMs for spheroid generation and application in regenerative medicine has been explored to prevent tumorigenicity and arrhythmias post-engraftment. Park et al. (2022) isolated contractile CD71^+^ hPSC-CMs and cultured them into spheroids ranging from 100 to 200 μm^29^. The survival and durability of these spheroids showed on average, twice the survival rate compared to single-CM monolayers under 3% hypoxic conditions, exhibited seven-day viability with 80% survival following six-month cryopreservation in liquid nitrogen. Additionally, improved cardiac function was achieved after their transplantation in an MI rat model, with reduced adverse remodelling of the left ventricular internal diastolic dimension and posterior wall thickness, as well as reduced fibrosis compared to other experimental groups^62^. These effects were mostly linked to the maturity observed in hiPSC-CM spheroid compared to single-cell transplants^29^. Reduced fibrosis due to cell-cell communication has previously been observed following the combination of hPSC-CMs with human bone marrow-derived mesenchymal stem cell-loaded patches^62^.

With the ongoing advances in tissue engineering, bioprinting has emerged as a state-of-the-art method for the de novo generation of spheroids using precise deposition of cell-laden materials, providing an avenue for the technique to be used in a clinical capacity once spheroids have been produced^30,63^. Using a method similar to the ‘Kenzan’ method^61^, Ong et al. (2017) utilised a 3D bioprinter to print CSs onto a needle array. Opting to print spheroids adjacent allowed the fusion and formation of spontaneously beating cardiac patches^18^. Others opting to generate CS containing patches using sodium alginate (Alg)-gelatin (Gel) hydrogels demonstrate recovery of cardiac function in MI mouse models^64^. Alg-Gel-based hydrogels are cost-effective, biocompatible and bioactive with viscoelastic properties that facilitate cardiomyocyte contractility^65^. The incorporation of silk fibroin, a naturally occurring polymer, has further improved the mechanical properties of Alg-Gel hydrogels, such as their elasticity and durability^66^.

On this note, while the development of novel hydrogels may be beyond the scope of this review, their potential to enhance the survival of exogenously transplanted cells in cardiovascular disease models warrants mention. Cytokine additives like SDF-1 in decellularized ECM hydrogels have been shown to promote cardiomyocyte survival in MI conditions, highlighting their potential therapeutic role^67^.

Roche et al. ^64^ compared three types of cardiac patches: i) acellular Alg-Gel hydrogels, ii) Alg-Gel hydrogels with freely suspended cardiac cells; and iii) Alg-Gel hydrogels containing CSs. Infarcted mice treated with the CS patches showed the best improvement in left ventricular ejection fraction % (LVEF%), which increased from 41% post-MI to 64% by day 28, nearing baseline function, an effect not observed with the other two types of patches. These results support that CS-containing patches could better restore heart function compared to acellular or freely suspended cell patches in humans. Additionally, transcriptomic analyses showed that gene expression profiles in mice treated with CSs were closer to non-infarcted (sham) profiles, supporting immunomodulatory effects facilitated by the transplantation of the spheroids. Overall, this study supports the potential use of CSs for myocardial repair *via* the immune response. In another study, Jeong et al. ^68^, demonstrated that the secretion of paracrine factors *via* the stromal cell-derived factor-1 (SDF-1$\alpha )$/CXCR4 axis induces cardioprotective effects on host cardiomyoblasts post CS implantation in MI mice models, and remodelling of the left ventricle^68^.

Preformed cardiac patches for implantation have also been explored by Noguchi et al.^13^. They cultured spheroids in a microarray and then transferred them to a gyrating, standard low-adhesive Petri dish. These spheroids were formed by the co-culture of rat neonatal ventricular cardiomyocytes, human dermal fibroblasts, and human cardiac microvascular endothelial cells^69^. Similarly, Mattapally et al. (2017) generated spheroids containing hiPSC-CMs embedded in a fibrin patch before engrafting them into infarcted mice. These presented a high ( > 25%) engraftment rate and an improvement in cardiac function, which they attribute to paracrine mechanisms. The conventional belief that spheroids exceeding 200 µm in diameter become susceptible to central apoptosis and necrosis was challenged by this work, as the group used spheroids that were 800 µm in diameter that did not develop a necrotic core^70^. Using a similar approach in a porcine-based MI model, Gao et al. (2018) employed CSs to generate larger heart patches (4 cm × 2 cm × 1.25 mm) that resulted in cardiac function improvement. Chansoria et al.^71^ recently generated a patch coated with extracellular vesicles derived from mesenchymal stem cells with wound-repair capability. Comprised of gelatin methacryloyl (GelMA), acrylic acid (ACA), calcium chloride (CaCl_2_), and curcumin nanoparticles, these patches demonstrated instant tissue adhesion (2.5-fold stronger than FDA-approved Tisseel), stretchability (>300% its original length without compromising elasticity), and rapid manufacturing (<2 min/patch). The patches synchronised with the diastolic and systolic cycles of the heart when transplanted into murine models^71^. It is worth mentioning that these studies follow previous trials that focussed on spheroid injection, supporting clinical safety and incorporation with tissue post-implantation in humans^72–76^.

### Disease modelling

The use of CSs for disease modelling and pathophysiology testing is not a new application. In fact, CSs have been used as models of ischaemic events^77,78^, congenital heart diseases^79^, and pregnancy-related cardiovascular disease^80^. Thanks to the use of induced pluripotent stem cells (iPSCs)^81–83^, it is possible to use patient-specific stem cells to generate CSs or similar 3D microtissues of non-spheroidal morphology that are specific to and compatible with a given patient^84,85^.

CSs have been applied as predictive risk-assessment models for pro-arrhythmic cardiotoxicity-causing agents^86,87^. When treated with ‘low-risk’ and ‘high-risk’ arrhythmia-inducing compounds, like E4031 and ranolazine, respectively, spheroids exhibit clinically expected outcomes^86^. Additionally, compounds like bisphenol A (BPA) and bisphenol F (BPAF), which induce pro-arythmic responses, are shown to cause contractile dysfunctions and cardiac hypertrophy in CSs, supporting their versatile application in drug discovery^88^. Cardiac spheroids have also been employed to model drug-induced toxicity associated with cancer therapies. For example, doxorubicin (DOX)-induced cardiotoxicity has been replicated in vitro using CSs, showing characteristic contractile dysfunction and cell death, along with changes in gene expression related to cardiac remodelling and fibrosis^89^. Protective effects of acetylcholine (ACh) against DOX toxicity were demonstrated using ACh-loaded nanoparticles, highlighting the role of nitric oxide signalling in endothelial cells as a potential cardioprotective mechanism^89^. In another study, ischaemia and ischaemia/reoxygenation (I/R) conditions were modelled in CSs by exposing them to altered oxygen levels. These CSs reproduced key features of myocardial infarction, including reduced contractility and expression changes in genes regulating sarcomere integrity, calcium handling, and remodelling^78^. This supports their use as physiologically relevant platforms for studying both I/R injury and chemotherapeutic cardiac damage.

Direct disease modelling of the C10orf71 gene, which is associated with dilated cardiomyopathy (DCM), has also been achieved in cardiac spheroids^90^. Following whole-exome sequencing of familial and sporadic DCM patients, frameshift mutations in hiPSCs were generated accordingly, then cultured into spheroids. C10orf71 ultimately contributed to reduced contractile function^90^. CS models exposed to normotensive or preeclampsia patient-derived plasma as a model of cardiovascular health showed a significant increase in FKBPL expression during late-onset of the disease, contributing to the emerging research into its predictive/diagnostic role in these patients^91^. CSs have been used to study the efficacy and potential mechanisms of action of guanxinning injections (used for coronary heart disease treatment). This study showed that the drug can inhibit cardiac fibrosis and hypertrophy through downregulation of the p38/c-Fos/Mmp1 axis, which mediates remodelling of cardiac tissue as well as protection against mitochondrial dysfunction in PE-induced hypertrophic spheroids^92^. Furthermore, CSs have been used to assess cardiovascular complications associated with COVID-19. Recent studies have shown that exposure of CSs to SARS-CoV-2 spike protein or plasma from COVID-19 patients leads to alterations in ACE2 expression, inflammation, and reduced beating frequency, mirroring clinical cardiomyopathies observed in COVID patients^93^. Altogether, these models support the versatility of cardiac spheroids and their broad applications in disease modelling and personalised medicine.

As testing on 3D in vitro tissues becomes more common, it may be that beyond CSs, tubular myocardial structures^85,94^, even whole beating miniature ventricles^95^, may provide enhanced (although increasingly complex and difficult to utilise) models for 3D tissue disease modelling. In one example, engineered heart tissue rings formed by injecting freshly isolated neonatal rat myocytes generated maximal twitch tensions of 2 mN/mm^2^ per contraction, which differs from the native heart tissue when we consider that this generates contractile tensions exceeding 20 mN/mm^2^ at rest and ~45 mN/mm^2^ at maximal output)^96–99^. In this context, maximal tension indicates the efficiency in contractility, and this is determined by the active and passive forces that influence the growth, morphology, and alignment of cardiomyocytes. To date, studies using human-derived iPSC-CMs showed that unaligned cardiosphere patches grown as discs (using 20 million cells/mL) exhibit contractile forces of 0.25 mN/mm^2^ compared to aligned strips, which measure at 0.6 mN/mm^2^ ^100^. Nevertheless, spheroids have been more broadly used than other models to create patient-specific tools for modelling complex pathophysiological processes, such as cardiac fibrosis^101^.

Considering the ability of spheroids to recapitulate the 3D microenvironment with few cells, their smaller size, large number of repeats, relative ease of use, and potential for scalable automation make them appealing alternatives to large constructs, and it is envisaged that this will have significant implications for both patient-specific and non-patient-specific disease testing^84,102^.

### Drug screening and cardiac safety testing

Accounting for both drug development failure and capital costs, bringing a drug to market has been estimated to cost approximately $879.3 million US dollars per drug and to take an average of 12–13 years^103,104^. In the USA, between 1980 and 2009, 3.5% of drugs approved for the market had to be withdrawn due to safety concerns that became apparent after the introduction of the drug to the market^105^. Among these, cardiac disorders appear as the second main reason (after hepatotoxicity), accounting for 18.8% of drug withdrawals in countries belonging to the World Health Organization^106^.

Against this backdrop, the appeal of novel 3D models that can model the effect of compounds on tissues in the laboratory in a biomimetic, reliable, and inexpensive way is evident. For example, testing of compounds on CSs presents the opportunity for early prediction and prevention of the hugely wasteful investment in drugs, which then must be removed from the market due to cardiotoxicity discovered at the end of the drug development process.

**Fig. 4:**
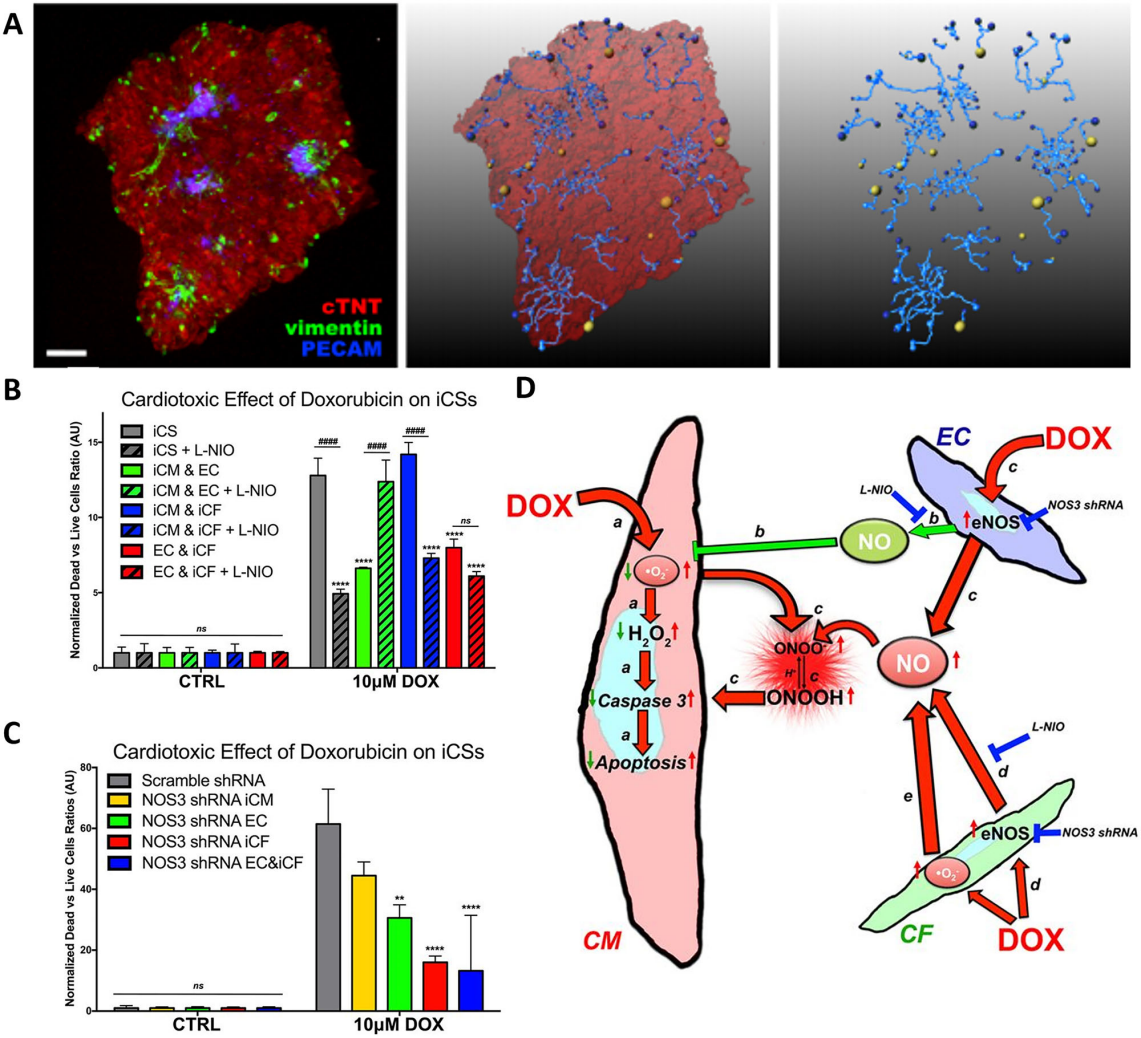
Cardiac spheroids as a model for drug toxicity assay. **A** Confocal microscopy (left panel) and 3D-rendered (central and right panels) images of a vascularised cardiac spheroid generated from iPSC-derived cells (iCS) stained with antibodies against markers for cardiomyocytes (red), cardiac fibroblasts (green) and endothelial cells (blue). Blue sticks follow the vascular network within the iCS. **B**, **C** Doxorubicin (DOX)-induced toxicity in iCS’s is dependent on endothelial nitric oxide synthase (eNOS). **B** Live/dead ratios of control iCS vs cardiac spheroids composed of two or three cell types exposed to 10uM DOX and L-NIO (a competitive antagonist of eNOS). DOX-induced toxicity is reduced in presence of L-NIO. **C** Live/dead ratios of control vs iCS where NOS3 is silenced in different cell types before treatment with 10 uM DOX. When compared to control (scramble) shRNA, DOX-induced toxicity is reduced when NOS3 is silenced in either endothelial cells or cardiac fibroblasts. **D** Schematic summarising the mechanisms of DOX-induced toxicity in iCS’s using chemical and genetic inhibitors (modified from ref. ^6^).

Evidence that 3D culture is superior to 2D for this purpose is mounting and is not just applicable to CSs^6,25,107,108^. For instance, 3D spheroids formed from mouse fibroblast L929 cells in HD cultures show greater resistance to doxorubicin, an anticancer molecule with cardiotoxic effects, than 2D cultures^107^. Further underscoring this relevance is the physiologically relevant ‘mini beating heart’ model developed by Polonchuk et al.^6^, which identified endothelial nitric oxide synthase as a key player in the mechanism of doxorubicin toxicity and confirmed that 2D monolayer models produced different results (Fig. 4).

One important driver for this bio-mimetic technology is the ability to test toxins and perform experiments where damage to cells is the primary endpoint^109^. Tomlinson et al.^108^, provide evidence that CSs reveal insights overlooked by monolayer cultures, as they lack intercellular crosstalk and cell-extracellular matrix interactions critical for testing compound effects. They demonstrate the cardioprotective effects of dexrazoxane—a cardioprotective agent co-administered with doxorubicin—using spheroids, along with other agents like NRF2 activators (bardoxolone-methyl and sulforaphane). This study illustrates the utility of CSs for complex multiple-compound testing^108^. Using spheroids, Barth et al.^110^ elucidated trastuzumab’s in vivo effects on human cardiac stem cells, which impair their ability to differentiate into cardiogenic cells and form microvascular networks^110^. Drug screening of a large pharmacologically diverse panel is also demonstrated using 15-FDA approved compounds associated with cardiotoxicity compared to 14-FDA approved compounds with no cardiotoxic association, showing evidence for improved specificity and comparable sensitivity when CSs are used versus monolayer cultures^111^.

Drug-induced cardiotoxicity remains a leading cause of compound withdrawal during development. Current regulatory assays fail to capture the complexity of arrhythmias or predict human responses accurately. Recent best practice recommendations advocate for iPSC-derived cardiomyocytes in 3D systems that may better replicate cardiac physiology^112^. This strategy promises to bridge the gap between receptor-based assays and human studies, offering a robust in vitro functional assay that improves clinical outcome prediction, minimises animal testing, and reduces costs.

### High-throughput assays and automation

To fully realise the potential of CSs as a transformative tool in drug testing, it is essential to develop high-throughput, scalable, and cost-effective platforms. Non-CSs of cancer cell lines in 384-well HD plates combined with automated liquid dispensing robots have already demonstrated the feasibility of high-throughput assays^25^.

In cardiac applications, Kouhry et al. (2021) optimised printing parameters for high-throughput CS array generation using a 3% (w/v) Alg–2% (w/v) Gel composition^58^. They utilised AC16 cardiomyocytes and adult human cardiac fibroblasts in a droplet-organoid model with advanced robotic systems. Recently, van Loo et al. ^113^ highlighted the start of the mass production of lumenogenic embryoid bodies and functional cardiospheres using in-air-generated microcapsules^113^.

A key challenge for high-throughput assays lies in automating data analysis. Progress is being made in this aspect, for example, by Sirenko et al., ^114^ who employed automated confocal microscopy and 3D Ca^2+^ oscillation assays using fast kinetic fluorescence imaging^114^. Clyde Bioscience’s CellOPTIQ® platform further integrates voltage, calcium, and contraction measurements in standard 96-well plates. Zuppinger^115^ described methods of quantitative video analysis and confocal microscopy that offer cost-effective solutions for contractility and calcium signal measurement without specialised equipment^115^.

Beyond optical methods, innovative technologies like 3D self-rolled biosensory arrays and organ-on-a-chip platforms are emerging. Kalmykov et al.^117^ demonstrated the utility of these arrays for studying cardiac electrophysiology, while Chikae et al.^116^ successfully validated 3D cardiac tissue chips constructed with a pin-type bioprinter^116,117^. These chips exhibited synchronised electrophysiological properties and drug-induced functional effects measured by calcium imaging and microelectrode arrays (MEA).

The versatility of iPSC-derived cardiomyocytes and other cardiac cells helps in developing complex in vitro models to facilitate systemic drug applications. Vascularised models represent a significant advancement, enabling the study of endothelial transport, tissue-specific uptake, and metabolic responses. Di Cio et al.^55^ utilised 96 ULA well plates to generate CSs composed of cardiomyocytes, endothelial cells, and fibroblasts in a 4:2:1 ratio, demonstrating consistent expression of contractile (e.g., cardiac troponin T) and cytoskeletal (myosin, F-actin, and α-actinin) markers indicative of strong vascular network stability^55^. Their vascularised heart-on-a-chip models demonstrated stable contractility and cytoskeletal marker expression, validating their utility in evaluating vandetanib-induced cardiotoxicity. Nonetheless, while iPSC-CMs-based models are increasingly used for myocardial tissue modelling, they may still fail at generating mature tissues, with reduced calcium and other ion channel expression^118^.

In order to evaluate contractile and electrical activity in spheroids, several attempts are reported. These include the use of an optical mapping system (MappingLab Ltd, Manchester, United Kingdom) for simultaneous recording of action potential and calcium imaging based on fluorescence signals^119^, as well as the use of microplate electrode array (MEA) plates, like the Maestro Pro (Axion Biosystems, Atlanta, USA) which offers scalable, label-free and automated reading in 96-well formats over days to months if required. For testing of beat-rate dependent drug effects, MEA plates can offer a significant advantage for high-throughput screening compared to optical mapping systems, which allow a limited number of recordings per time. Additionally, protocols using optical mapping systems may require additional optimisation and may not be as intuitive as using an MEA plate. Therefore, future considerations around better ways to control and measure electrical activities in spheroids might be required. In one attempt, magnetic gold-iron oxide nanoparticles were added to spheroids to prevent them from free-floating in wells and therefore to reduce signal-to-noise ratios in the Maestro Pro system^118,120^. Despite ongoing optimisations, these advances underscore the promise of CSs for innovative drug development, offering precise, scalable, and ethical testing solutions that bridge preclinical research gaps and enable translation to the clinic.

## Discussion and future perspectives

Whilst it is increasingly evident that CSs represent an important advance from monolayer techniques, every system has advantages and disadvantages, and monolayer techniques may remain preferable in certain situations. For example, Bursac et al.^121^ argue that, when studying electrical propagation, monolayers provide certainty that the entire signal is produced from only one layer of cells, which allows for direct comparison to computer simulations and 2D theoretical models^121^. Not to mention, single-cell testing can also yield important insights, as illustrated by a recent study of patient and disease-specific cells using iPSC-CMs derived from skin cells of a patient with Brugada syndrome and the SCN10A mutation^122^. Though the chosen experimental model was effective for investigating single-cell phenotypes and single-cell ion channel behaviour, it failed to account for more complex cell-cell interactions as well as hormonal and neural regulating factors.

However, for a truly comprehensive study of cardiovascular diseases in 3D, more physiologically relevant tissue constructs are required. These can be achieved through fusion in rows^100,123^, filamentous fibres mimicking heart muscle^124–126^, larger constructs such as slices of heart tissue cut with a microtome^127^, 5 mm diameter discoids^14^, doughnut-shaped engineered heart tissue^128–130^, or even miniature whole heart chamber models^95^. Indeed, high-throughput techniques including automated analysis of Ca^2+^ transients have been described in ring-contractile 3D-engineered heart tissue with an inner diameter of 2 mm, an outer diameter of 4 mm, and a height of 5 mm^131–133^. Cylindrical trabecular strips of heart tissue, as in the ‘Biowire II’, also show the need to generate robust long-term models for chamber-specific disease cases like left ventricular hypertrophy, which implicates chronic increased workloads on cardiac tissue over months^125^.

Despite all the advancements and protocols described in this review article around CSs, it is important to highlight some of the current limitations, which include elevated costs (especially in case of patient-derived stem cells), scalability (limited by the access to enough cells to produce the required number of spheroids, as well as access to specialised equipment that may allow this), and batch variability. The latter is particularly important when considering personalised CSs, where patient-specific cells will be used and therefore standardisation of the production and use might require further considerations and optimisation.

To date, the full recapitulation of the cardiac microenvironment is limited, even in spheroid cultures, no studies have included all eleven resident and non-resident cell types present in the heart. To overcome this challenge, biomaterials might be used to some extent, which results in limited cardiac function in vitro. For instance, electrical conductivity is an inherent feature of the myocardium that promotes synchronous contractility^134^. Spheroids generated by combining hiPSC-derived cardiomyocytes with biocompatible conductive polymers like silicon nanowires (at a 1:1 ratio) show enhanced sarcomeric alignment, elevated expression of electroconductive transcripts (e.g., Cx-43, sarcomeric-α, β-myosin heavy chain, and N-cadherin), and improved CS maturation and beating^135–137^. Nevertheless, a potential future area of research using spheroids may focus on recapitulating metabolic disorders linked to congenital heart defects. Additionally, new models of cardiogenesis could be generated following recent studies that focussed on the use of controlled doses of small molecule inhibitors (e.g., Wnt-C59) and activators (*e.g*., CHIR99021) of Wnt signalling^138^. Despite the interchangeable nomenclature between spheroids and organoids, Wnt-driven cardiogenesis more accurately represents cardioids and organoids, based on their advanced tissue architecture showing stable chamber formation with well-defined endocardial and epicardial layers^138,139^. Of note, cardioids emerged as self-organised organoids from human pluripotent stem cells using key cardiogenic signalling pathways—ACTIVIN, bone morphogenic protein (BMP), fibroblast growth factor (FGF), retinoic acid (RA), and WNT^139^.

The aforementioned limitations do not preclude further investigation or utilisation of CSs for precision medicine—rather, as discussed in relation to hydrogels, modifying the cardiovascular niche itself with cardiac-tailored biomaterials may facilitate the engraftment of spheroids within localised infarcted regions in a patient-specific approach. However, given the extensive in vivo testing and regulatory approvals required, use of cardiac spehroids for precision medicine in vivo may require additional time. Nevertheless, CSs provide a sophisticated platform for drug screening and cardiotoxicity testing with the potential to aid in the prediction of treatments for patients stratified into high heart disease risk groups.

Besides spheroids, it is important to remember that alternative 3D models could be used, which also present advantages and disadvantages. For instance, thin cardiac slice models retail the adult tissue architecture and multi-cell type complexity of the tissue from which they are derived, making them compelling to use over spheroids, organoids, organ-on-chip or 3D bioprinting methods. These have been used particularly in clinical settings for the management of myocardial diseases (e.g., cardiac sarcoidosis, immune checkpoint inhibitor myocarditis, or heart transplant rejection)^140^. However, they may not fully capture the whole tissue structure as they are sliced < 400 μm and are cultured on a cell culture electrical simulator (e.g., IonOptix, C-Pace EP Cell Culture Simulator) to continuously stimulate them at 1–1.2 Hz^141,142^. Protocols improving culture conditions through timed media changes that promote adequate oxygen supply show that functional viability can be maintained up to 6 days with transcriptionally normal gene expression profiles^143^. Contrasting with the month-long cultures of spheroids, this culture duration is a barrier that makes thin slices less scalable and limits their uses for acute and subacute cardiotoxic therapeutic testing, rather than chronic^141^. Nonetheless, perhaps the biggest limitation of thin slice models is the need for a recurrent tissue supply, which, compared to sophisticated cell-based methods, is not a hindering limitation as cells and consumables are more readily available. Eventually, the purpose and the mechanistic question will determine the model to be utilised in specific studies, alongside the appropriate governing bodies [U.S. Food and Drug Administration (FDA) and the European Medicines Agency (EMA)] white paper, which is co-authorised by industry and academic experts to outline the general validation principles for the models^144^.

Despite growing interest in spheroid research, their translation into clinical applications is not without challenges, particularly in meeting regulatory standards for therapeutic efficacy, uniformity, and universal reproducibility. This is reflected in a limited number of published studies and registered clinical trials using CSs for regenerative applications. A notable trial, currently recruiting, is the HS-001 phase I/II study, which will be reporting on the safety and efficacy of transplanting hiPSCs-derived cardiomyocyte spheroids in severe heart failure patients who have received screening less than a month post a myocardial infarction (ClinicalTrials.gov, 2025, NCT04945018). This trial was possible due to preclinical validation of intramyocardial transplantation as a delivery method using hiPSC-derived CSs in mouse and swine heart failure models^145^. The preclinical study showed that, in rodent and swine models, transplanted spheroids can reduce infarct zones without enhancing cardiac hypertrophy and angiogenesis^145^. Central to their work was the optimisation of their culture methods, allowing for the isolation of cardiomyocytes with high purity. Following a comparison of metabolic pathways between cardiomyocytes and iPSCs, the group developed a culture system that eliminates undifferentiated iPSCs and non-cardiomyocyte cells that can contribute towards teratoma formation if engrafted^146^.

The establishment of brief universal definitions that characterise the primary difference between CSs and organoids may also be beneficial to aid better documentation of their respective generation methods. One simple way could be to define CSs as self-organising cellular aggregates that exhibit a simple rounded morphology, while cardiac organoids can be defined as self-organising cellular aggregates with self-renewal capability and the ability to develop intricate morphologies with defined structures, more closely resembling the complex microenvironment of the native heart. While this definition might attract the most consensus at the moment, it is important to highlight that engineered heart tissues and other models have also been referred to as organoids, creating additional confusion in the field. A clear definition, such as the one provided here, can prevent further confusion and will facilitate the standardisation of methods used to generate CSs for clinical applications.

Additional considerations that can facilitate the use of CSs in the clinic include the use of non-animal-derived products in culture to avoid immune responses as well as to facilitate regulatory approvals, and the need for stringent assessment over the manufacturing process, with strict quality assurance protocols to ensure patient safety as well as efficiency and uniform interpretations across clinical settings.

## Conclusions

Replicating the native myocardium in vitro requires a replacement of standard 2D cell culture with advanced concepts in a multidisciplinary approach comprising tissue engineering, biomaterial science, and cardiomyocytes. By bridging these diverse areas, CSs open new avenues in cardiac regeneration, disease modelling, and compound testing. As in vitro models, CSs are well-suited for testing patient-specific or disease-specific conditions. CSs can be generated using a variety of methods, either using one of them or a combination of a few of them. This will be directed by the specific application of the CS culture. Several advantages and disadvantages remain in their use, which may prompt novel venues of research areas to improve their scalability, cost, and reproducibility. Further considerations regarding regulatory pathways will also facilitate their translation from the bench to the bedside for cardiovascular disease patients. In the future, it is envisaged that CSs combined with scalable automatic techniques will dramatically change the way cardiac tissues can be engineered, cardiac diseases can be modelled, and cardio-active compounds can be tested.

## Data Availability

No datasets were generated or analysed during the current study.
